# Nanoformulation of Azadirachtin Improves Its Control on Cotton Pests

**DOI:** 10.3390/molecules31132347

**Published:** 2026-07-03

**Authors:** Zhiwei Tang, Jianhao Dong, Yue Sun, Chuhela Tabusibieke, Yujiao Wang, Wei Lu

**Affiliations:** 1 Key Laboratory of the Pest Monitoring and Safety Control of Crops and Forests of the Universities of the Xinjiang Uygur Autonomous Region, College of Agronomy, Xinjiang Agricultural University, No. 311, Nongda East Road, Sayibak District, Urumqi 830052, China; 18320027289@163.com (Z.T.); dong371014@163.com (J.D.); 15709013626@163.com (Y.S.); c21521sahpr@foxmail.com (C.T.); 2 Engineering Research Centre of Cotton, Ministry of Education, Urumqi 830052, China

**Keywords:** azadirachtin, mesoporous silica nanoparticles, *Helicoverpa armigera*, *Apolygus lucorum*

## Abstract

The practical efficacy of Azadirachtin (AZA)—a botanical insecticide valued for its strong antifeedant activity—is often undermined by its poor environmental stability, arising from rapid photolysis and thermal decomposition. Herein, amino-modified mesoporous silica nanoparticles (MSNs) were employed as nanocarriers to encapsulate AZA, yielding the AZA@MSNs−NH_2_ nanoinsecticide. Under UV irradiation (254 nm), the degradation rate of AZA@MSNs−NH_2_ was more than 35% lower than that of commercial AZA after 24 h of exposure. In biosafety tests, MSNs at 50–800 mg/L enhanced cotton seed germination and seedling growth, whereas MSNs below 200 mg/L caused no observable harm to zebrafish survival. Free AZA (0.5–10 mg/L) showed limited efficacy in bioassays against *Helicoverpa armigera* and *Apolygus lucorum*, reducing larval weight gain only marginally in the former (mortality < 5%) and performing poorly against the latter. In contrast, AZA@MSNs−NH_2_ significantly boosted both growth inhibition and lethal effects against both pests, yielding > 80% growth inhibition and 62.65% corrected mortality for *H. armigera*, as well as strong lethality toward *A. lucorum*. Overall, this work confirms that nanoformulation of AZA not only circumvents its physicochemical drawbacks but also boosts its insecticidal performance against cotton pests, supporting the advancement of sustainable cotton pest control.

## 1. Introduction

The Xinjiang Uygur Autonomous Region is renowned as China’s leading cotton-producing area, producing high-quality fiber that dominates domestic textile supply. Yet, sustainable pest management practices—including those relying on botanicals like azadirachtin (AZA)—have proven inadequate against recurring infestations of *Apolygus lucorum* and *Helicoverpa armigera* [1,2]. Chemical insecticides remain the dominant approach for pest management in cotton cultivation [3]. It is widely recognized that conventional synthetic insecticides, characterized by their low degradability, contribute to pollution of soil, water, and air, while posing potential health risks to humans through bioaccumulation [4]. Botanical insecticides, by contrast, present notable benefits including high bioactivity, eco-friendliness, and renewability [5], making them more compatible with sustainable agriculture [6]. Moreover, they exhibit minimal toxicity to natural enemies and crops, and rarely trigger pest resistance. Their commercialization and field utility, however, remain constrained by elevated production expenses and inadequate photochemical/thermal tolerance [7,8].

Azadirachtin (AZA), one of the most potent insect antifeedants globally [9,10], is a complex tetracyclic triterpenoid with robust insecticidal activity extracted from the seeds and leaves of the neem tree (*Azadirachta indica*) [11,12]. It primarily targets core pathways regulating insect growth and development [13], and exhibits strong toxicity against major agricultural pests such as *Plutella xylostella* and *H. armigera* [14,15,16,17]. Compared with synthetic insecticides, AZA degrades rapidly [15], generates no residual accumulation in soil and imposes markedly milder adverse effects on non-target taxa including birds, mammals, and bees [8]. However, AZA possesses low aqueous solubility and thus demands organic solvents for complete dissolution [11]. Furthermore, it is highly susceptible to light and heat, factors that readily trigger photolytic and thermal degradation under field conditions [18,19,20].

Formulation optimization, especially nanotechnology-enabled modification, offers a viable route to overcome the intrinsic limitations of AZA. Iqbal et al. [21] for example, developed AZA-loaded nanoemulsions (NEs) by solubilizing the hydrophobic compound in oil cores, and with natural bioadjuvants, they improved droplet spreading and retention, leading to stronger activity against *Bemisia tabaci* (Gennadius, 1889) [21]. Nevertheless, NEs are inherently liquid dispersions, which fail to provide a protective solid barrier; consequently, the encapsulated AZA remains vulnerable to photodegradation at the oil–water interface. Furthermore, the lack of a rigid matrix often compromises their physical stability, causing premature release and shortened residual activity. Separately, Pascoli et al. [22] prepared neem oil-loaded zein nanoparticles from maize, but their study stopped short of biological validation, as neither laboratory bioassays nor field efficacy tests were conducted to substantiate the insecticidal potential of these carriers [22]. These two significant gaps—namely, the inherent instability of liquid formulations and the absence of biological efficacy data—highlight the pressing need for a solid carrier system with demonstrable insecticidal performance against key cotton pests.

At present, nanocarriers have emerged as a pivotal strategy for upgrading pesticide formulations owing to their capacity to encapsulate active ingredients [22,23,24], mitigate ecological toxicity, and boost pesticide stability and utilization efficiency [25,26]. Among diverse carrier substrates, mesoporous silica nanoparticles (MSNs) feature large specific surface areas, straightforward synthetic routes, tunable particle sizes, and favorable aqueous dispersibility [27,28]. Furthermore, MSNs act as superior nanocarriers with adjustable pore sizes, homogeneous pore distribution and robust structural stability [29,30,31,32]. Accordingly, MSNs were chosen as the carrier material in the present work [33].

A variety of synthetic routes for MSNs have been established [34], including top-down approaches (mechanical ball milling, etc.) or bottom-up approaches (the classic hydrothermal method, Stöber method, template method, and Sol–Gel method), with the Sol–Gel route being the most prevalent due to its simplicity, ease of handling, and cost-effectiveness [35,36]. Furthermore, the Sol–Gel method and its variants (e.g., modified Stöber) are currently the most established and industrially scalable routes, offering the flexibility to tune particle size, porosity, and surface functionality of MSNs simply by varying reactant ratios, temperature, or template choice—thereby accommodating pesticides of diverse molecular sizes and charge characteristics.

Studies have demonstrated that alkaline reagent dosages and distinct template removal protocols exert significant impacts on the particle size, pore structure, and dispersity of as-synthesized MSNs, thereby further regulating their subsequent pesticide loading capacity [37]. Regarding surface modification tactics, amino functionalization serves as a simple, rapid, and high-efficiency approach that can markedly elevate the loading capacity of nanocarrier substrates [38,39].

To address the aforementioned challenges, we first optimized the synthesis parameters of MSNs, with particular emphasis on NaOH concentration and the choice of template removal strategy (refluxing vs. calcination). The optimized nanocarriers were subsequently functionalized with amino moieties, which imparted improved hydrophilicity, enhanced dispersion stability, and favorable surface charge characteristics—features that synergistically promoted high-efficiency AZA encapsulation [40,41]. Using these optimized MSNs as nanocarriers, an AZA nanoformulation (AZA@MSNs−NH_2_) was constructed and its insecticidal activity was systematically assessed against *H. armigera* and *A. lucorum*. These findings are expected to provide both empirical support and theoretical groundwork for the development of eco-friendly pest management in cotton production.

## 2. Results and Discussion

### 2.1. Synthesis, Amino Modification and Physicochemical Characterization of MSNs

#### 2.1.1. Removing Templates

**Figure 1:**
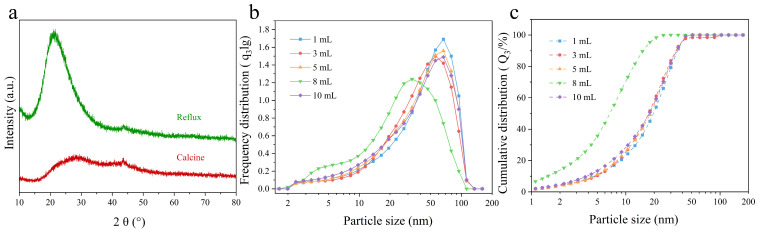
XRD patterns (**a**), particle size frequency distribution (**b**), and cumulative particle size distribution (**c**) of the synthesized MSNs.

According to the XRD results, MSNs fabricated via the two template removal methods display a characteristic diffraction peak at approximately 2θ = 23°, consistent with the typical XRD profile of amorphous silica (Figure 1a) [42]. No distinct impurity peaks appear in the diffractograms, confirming that both protocols yield phase-pure MSNs (Figure 1a). However, the characteristic peak of calcined MSNs is broader relative to reflux-treated samples, indicative of decreased structural ordering and a loosely condensed silica network [43,44]. Given that a well-condensed silica structure is critical for maintaining carrier integrity, the reflux method was therefore selected for template removal in subsequent MSNs synthesis.

#### 2.1.2. Nitrogen Adsorption–Desorption and Pore Size Distribution Profiles of MSNs and AZA@MSNs−NH_2_

As shown in Figure S1a,b, both materials exhibit typical Type IV nitrogen adsorption–desorption isotherms consistent with IUPAC classification. For pristine mesoporous silica nanoparticles (MSNs, Figure S1a), a sharp increase in nitrogen uptake appears at a relative pressure (P/P_0_) of 0.3–0.9, accompanied by a distinct H1-type hysteresis loop. This reveals that MSNs possess highly ordered, homogeneous mesoporous channels. The corresponding pore size distribution presents a narrow unimodal curve centered at 3–4 nm, confirming the well-organized mesostructure of the as-synthesized silica.

After azadirachtin (AZA) loading (Figure S1b), the mesoporous framework of the composite material remains intact. Nevertheless, the notable drop in nitrogen adsorption capacity, together with shifts in the position and shape of the hysteresis loop, verifies successful AZA immobilization inside the mesoporous channels. Furthermore, the reductions in specific surface area, average pore diameter, and total pore volume further confirm efficient encapsulation of AZA molecules within the pore network of MSNs.

#### 2.1.3. The Addition Amount of Sodium Hydroxide Solution

The dosage of alkaline reagent used during MSNs synthesis affects the hydrolysis rate of the silicon precursor, mediates nanoparticle generation, and consequently tunes the final particle size and particle size distribution [37].

In this study, different volumes (1, 3, 5, 8, and 10 mL) of 2 mol/L NaOH solution were introduced into the reaction system, and the particle size and distribution uniformity of the as-synthesized MSNs were characterized. Particle size analysis (Figure 1b,c) showed that MSNs synthesized with 1, 3, 5, and 10 mL NaOH solutions possessed larger average particle diameters ranging from 42.66 nm to 87.53 nm. In contrast, the sample prepared with 8 mL of NaOH displayed the minimum average particle size (22.71 nm) along with the narrowest size distribution. Previous investigations have demonstrated that when TEOS undergoes full hydrolysis with sufficient surfactant present, MSN particle size rises as the dosage of alkaline reagent increases [37,45].

Notably, these findings differ from previous reports, which may be attributed to the inability to sustain a sufficiently strong alkaline environment at excessively low NaOH concentrations under the present experimental conditions [39]. Under such circumstances, TEOS only undergoes partial rapid hydrolysis to form poorly soluble condensates and primary particles, ultimately yielding fewer, larger nanoparticles. Conversely, a high NaOH concentration promotes rapid TEOS hydrolysis, yielding primary silica particles with high surface free energy. The limited surfactant in the reaction system is incapable of fully coating all generated nuclei and effectively lowering their surface energy, which inevitably leads to interparticle aggregation and the emergence of larger-scale architectures [39].

#### 2.1.4. Amination Modification of MSNs and Characterization of MSNs–NH_2_

**Figure 2:**
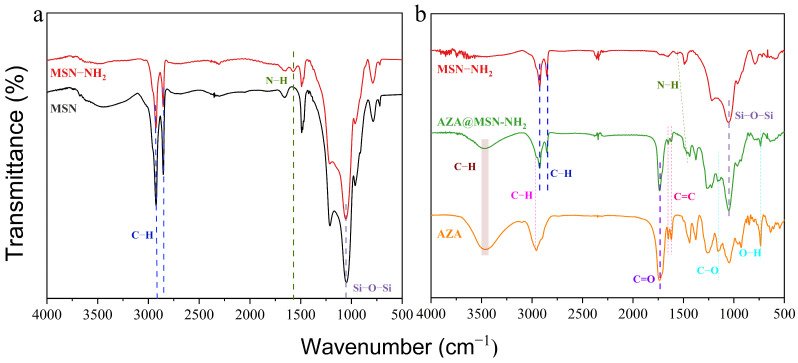
FT-IR spectra of MSNs and MSNs–NH_2_ (**a**), AZA and AZA@MSNs–NH_2_ (**b**).

The FTIR spectrum of bare MSNs (Figure 2a) displays characteristic absorption bands at 2920 cm^−1^ and 2857 cm^−1^ (C–H stretching vibration) as well as 1048 cm^−1^ (asymmetric Si–O–Si stretching vibration), confirming the successful synthesis of MSNs [46,47]. To boost the AZA loading capacity, MSNs were modified with amino groups to yield MSNs–NH_2_. The FTIR spectrum of MSNs–NH_2_ (Figure 2a) maintains all characteristic peaks of pristine MSNs and presents an extra band at 1560 cm^−1^ corresponding to N–H bending vibration [48,49], which verifies complete amino functionalization.

#### 2.1.5. The Zeta Potential of MSNs and AZA@MSNs–NH_2_

The surface charge properties of pristine MSNs and AZA@MSNs–NH_2_ were characterized by zeta potential measurements (Figure S2). Pristine MSNs displayed a highly positive zeta potential of 45.3 mV, which dropped to 29.9 mV following AZA loading. This elevated initial potential is primarily attributed to the retention of cationic CTAB on the MSN surface, as the alkaline synthetic environment favors the stability of CTAB micelles, rendering them difficult to eliminate completely by conventional washing. AZA, being a neutral tetracyclic triterpenoid lacking ionizable groups [50,51], does not contribute to charge compensation. Therefore, the observed potential decrease is reasonably explained by the gradual removal of residual CTAB and the partial shielding of positively charged surface sites during the repeated centrifugation/washing cycles employed in the loading process.

### 2.2. Optimization of AZA Loading

#### 2.2.1. Loading AZA with Unmodified MSNs

In view of the facile synthesis route, AZA was loaded onto bare MSNs (AZA@MSNs) and evaluated by LC and FT-IR. Regrettably, the AZA payload on bare silica carriers proved to be negligibly low, falling below the quantitation limit of LC, while the FT-IR spectrum exhibited no detectable vibrational features attributable to AZA (Figure S3). These observations collectively indicate that pristine MSNs lack sufficient surface affinity for the nonionic AZA molecule, underscoring the necessity of surface amino modification to improve drug loading.

#### 2.2.2. Loading AZA with MSNs–NH_2_

To enhance AZA loading efficiency, MSNs were functionalized with an amination reagent. AZA possesses hydrogen bond donor moieties (–COOH) and can be loaded onto MSNs–NH_2_ via hydrogen bonding with surface –NH_2_ groups of the modified carrier, which act as hydrogen bond acceptors. The preparation procedure was optimized with reference to the study by Benítez et al. [34], to construct mesoporous composites relying on hydrogen-bonding interactions, thereby improving the encapsulation efficiency of AZA.

**Figure 3:**
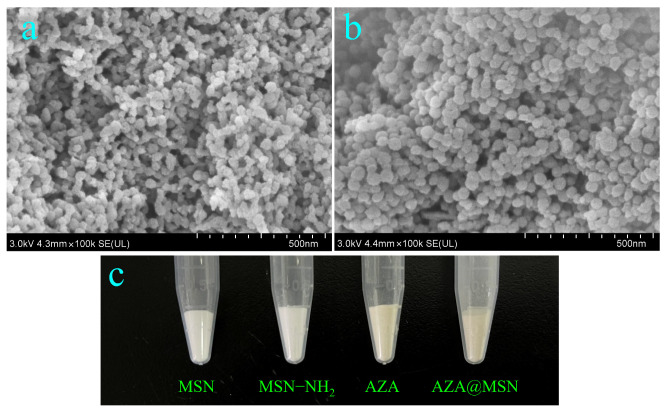
SEM micrographs of MSNs–NH_2_ (**a**) and AZA@MSNs–NH_2_ (**b**) and digital photographs of MSNs, MSNs–NH_2_, free AZA and AZA@MSNs–NH_2_ (**c**).

As shown in Figure 3b, the FT-IR spectrum of AZA@MSNs–NH_2_ displays characteristic bands originating from both MSNs–NH_2_ and free AZA: unsaturated and saturated C–H stretching vibrations at 3460 cm^−1^ and 2950 cm^−1^, C=O stretching at 1732 cm^−1^, C=C stretching at 1650 cm^−1^ and 1620 cm^−1^, C–O stretching at 1152 cm^−1^, and O–H out-of-plane bending vibration at 733 cm^−1^. In addition, due to hydrogen bond formation, the N–H stretching vibration of bare MSNs–NH_2_ at 1560 cm^−1^ shifts to around 1462 cm^−1^ in the spectrum of AZA@MSNs–NH_2_ [52]. These results confirm the successful loading of AZA.

According to SEM micrographs of MSNs–NH_2_ and AZA@MSNs–NH_2_ (Figure 3a,b), the originally rough surface of MSNs–NH_2_ nanoparticles turns markedly smoother after AZA loading, whereas the particle size barely changes. As presented in Figure 3c, MSNs and MSNs–NH_2_ exist as white powders; by contrast, pure commercial AZA and AZA@MSNs–NH_2_ show bright yellow and dark yellow appearances, respectively.

As presented in Figure S4a, bare MSNs display monodisperse spherical morphology with narrow size distribution and favorable colloidal stability, only accompanied by minor aggregation. These nanoparticles possess distinct edges and smooth surfaces free of obvious surface coatings, consistent with typical conventional mesoporous silica nanostructures. By contrast, Figure S4b reveals aggregated composite architectures. Such aggregation originates from hydrogen bonding and electrostatic interactions derived from surface amino moieties, which trigger interparticle crosslinking and generate irregular surface coatings. A notable rise in overall particle size occurs alongside a morphological transition from smooth to rough surfaces. The ring-shaped dark domains on particle surfaces indicate the coexistence of amino-modified layers and adsorbed AZA, offering direct morphological evidence of successful amination and AZA immobilization. Referring to the research of Benítez et al. [34], we grafted amino groups onto MSNs to enhance the aqueous dispersibility of both pristine MSNs and the AZA@MSNs–NH_2_ composite.

**Figure 4:**
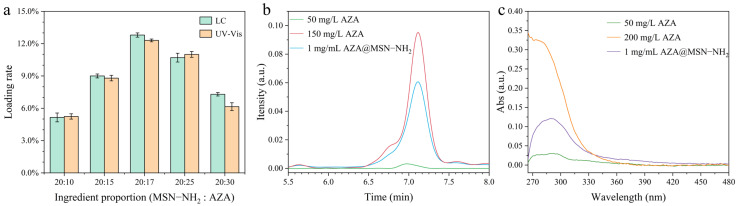
AZA loading capacities under different feed mass ratios (**a**), LC chromatogram (**b**), and UV–Vis spectrum of AZA@MSNs–NH_2_ with the maximum loading capacity (**c**).

The feeding mass ratio of AZA to MSNs–NH_2_ was optimized, and the AZA loading efficiency was determined by LC and UV-Vis spectroscopy, with the respective standard curves presented in Figure S5a,b. As shown in Figure 4a, when the feeding ratio of AZA to MSNs–NH_2_ was 20:17, the AZA loading capacity reached a maximum of 12.8% (determined by LC) and 12.3% (determined by UV-Vis). The LC and UV-Vis spectra of AZA@MSNs–NH_2_ with the highest loading rate are displayed in Figure 4b,c. Under the optimal preparation condition for AZA@MSNs–NH_2_, the encapsulation efficiency was calculated to be 10.6% according to the formula in Section S1.3 of Supplementary Materials.

### 2.3. Resistance to Photodegradation by UV Light

**Figure 5:**
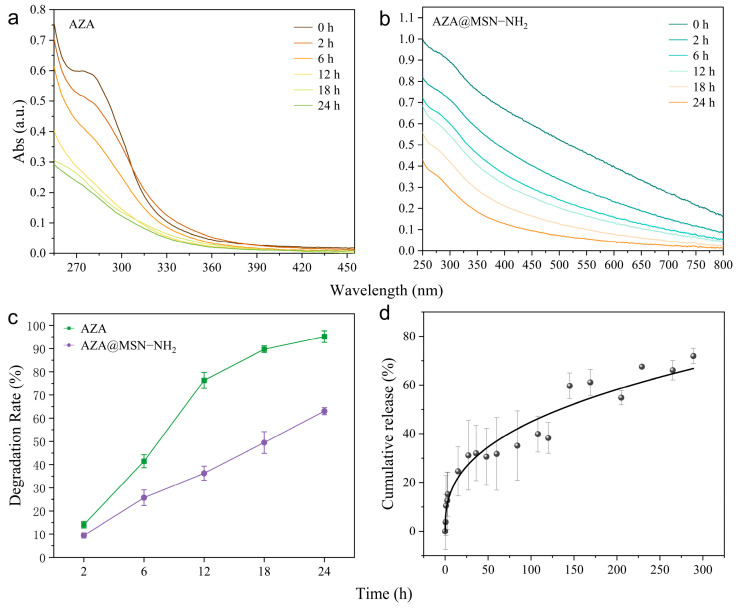
UV–Vis spectra of free AZA (**a**) and AZA@MSNs–NH_2_ (**b**), photodegradation rates under UV irradiation (**c**) of free AZA and AZA@MSNs–NH_2_, and cumulative release profiles (**d**) of AZA@MSNs–NH_2_.

Numerous studies have demonstrated that encapsulation within MSNs can effectively mitigate the photodegradation of loaded pesticides [53,54]. Accordingly, the photostability of AZA@MSNs–NH_2_ was characterized in the present study. Degradation curves of free AZA and AZA@MSNs–NH_2_ under 24 h UV irradiation are presented in Figure 5a,b. As observed, nearly half of pure AZA degraded after 6 h of UV exposure. After 24 h irradiation, AZA’s characteristic UV absorption band near 274 nm nearly vanished, corresponding to a degradation percentage of 95.18 ± 2.47%.

In comparison, AZA@MSNs–NH_2_ displayed greatly inhibited photodegradation, with degradation values of merely 25.76 ± 3.33% and 63.00 ± 1.53% after 6 h and 24 h irradiation, respectively (Figure 5c). Furthermore, the prominent absorption band at around 274 nm remained strong in the spectrum of AZA@MSNs–NH_2_. Consistent with the findings reported by Beníte et al. [34], our work verifies that MSNs remarkably enhance the photostability of AZA. Fabrication into the AZA@MSNs–NH_2_ nanoinsecticide drastically lowers AZA’s vulnerability to photolysis and greatly elevates its light stability.

The sustained-release behavior of AZA@MSNs–NH_2_ was evaluated to clarify the protective role of MSNs–NH_2_ toward AZA. As shown in Figure 5d, AZA@MSNs–NH_2_ exhibited a gradual release trend: cumulative AZA release rose rapidly at the early stage and slowed down afterwards. Roughly 30–40% of encapsulated AZA was released within 48–72 h, followed by continuous slow release reaching nearly 70% at 288 h. Such a release profile confirms that AZA molecules are confined within mesoporous channels and diffuse outward gradually, matching the tailored biphasic release characteristic of azadirachtin loaded in organically modified mesoporous silica nanocarriers reported by Jokarshourijeh et al. [51]. The controlled-release feature of AZA@MSNs–NH_2_ reduces direct UV exposure of AZA payloads, which accounts for enhanced anti-photodegradation performance and extended insecticidal bioactivity.

### 2.4. Biosafety Assay of MSNs

AZA is a green and eco-friendly bioinsecticide. Biosafety assessments were carried out to verify that the AZA@MSNs–NH_2_ formulation retains this environmentally benign property. We evaluated the impacts of blank MSNs on wheat seed germination, cotton seedling growth, and zebrafish survival, with detailed results displayed as follows.

#### 2.4.1. The Effects of MSNs on Cotton Seeds and Seedlings

As shown in Table S1, although cotton seed germination rates under different MSNs concentrations showed no statistically significant differences relative to the control group, all groups presented numerically elevated values with a concentration-dependent upward trend. Moreover, germination potential and seedling height of cotton seeds in all MSNs treatment groups surpassed those of the control. Notably, MSNs concentrations from 50 to 800 mg/L induced remarkably higher indicators compared with the control. These observations reveal that MSNs within a proper concentration range facilitate cotton seed germination and seedling development.

As displayed in Figure S6a,b, cotton seeds exposed to high MSNs concentrations contained substantially higher levels of abscisic acid and gibberellin than the control, further confirming that excessive MSNs exert inhibitory effects on cotton seed germination. Figure S6c illustrates that the α-amylase activity of cotton seeds first rises and then gradually declines with elevated MSNs concentrations. As shown in Figure S6d, moderate MSNs concentrations markedly accelerated protein hydrolysis in cotton seeds, while high concentrations slightly suppressed seed germination.

#### 2.4.2. The Effects of MSNs on Wheat Seeds and Seedlings

As shown in Table S1, wheat seed germination and seedling growth were significantly promoted at 100 mg/L MSNs relative to the control. However, at concentrations of 200 mg/L and above, both parameters were markedly inhibited, with the germination rate dropping to only 50% at 800 mg/L. Regarding germination potential, treatments up to 200 mg/L maintained values above 70%, comparable to the control, whereas concentrations of 500 mg/L and above caused a significant reduction, falling to just 25% at the highest tested dose (800 mg/L). These results indicate that MSNs exhibit a hormesis-like effect at low concentrations (100 mg/L), establishing a safe concentration window for their application as nanocarriers in agricultural settings.

The physiological basis for MSNs-induced phytotoxicity was further investigated by measuring key enzyme activities and hormone levels in treated wheat seeds (Figure S6). High-concentration MSNs treatments significantly elevated the contents of both ABA and GA (Figure S6a,b), consistent with their role in retarding germination. Concurrently, α-amylase activity exhibited a dose-dependent decrease (Figure S6c), which would limit the breakdown of starch reserves and constrain energy supply. Conversely, trypsin activity showed a dose-dependent increase (Figure S6d), likely reflecting a stress-induced enhancement of proteolytic capacity to mobilize nitrogen reserves for seedling establishment. Despite this potential compensatory mechanism, the combined impact of hormone imbalance and suppressed carbohydrate metabolism leads to an overall inhibitory outcome on germination at elevated MSNs concentrations.

Sun et al. [55] reported that MSNs exert no effect on wheat seed germination while promoting post-germination seedling growth and photosynthesis. The results of this study, however, reveal a more nuanced, concentration-dependent pattern: MSNs at 100 mg/L significantly promoted germination, whereas concentrations ≥ 200 mg/L inhibited both germination and subsequent seedling development. Specifically, root and leaf lengths were markedly reduced at ≥200 mg/L, and seedling height decreased significantly at ≥500 mg/L. Collectively, these findings demonstrate a biphasic dose–response—low-dose stimulation coupled with high-dose inhibition—underscoring the importance of dosage optimization when applying MSNs as agricultural nanocarriers.

#### 2.4.3. The Influence of MSNs on *Danio rerio*

The acute toxicity of MSNs to zebrafish was dose-dependent over a 96 h exposure period (Figure S7). At concentrations ≤ 200 mg/L, no mortality was observed, and even at 500 mg/L, the survival rate was statistically comparable to that of the control, confirming the biosafety of MSNs within this concentration range. Toxicity became evident, however, at higher concentrations: at 800 mg/L, survival decreased progressively from 71.11 ± 3.85% at 24 h to 48.89 ± 3.85% at 96 h; at 1000 mg/L, survival plummeted to 51.11 ± 10.18% within 6 h, with complete mortality by 96 h. The sharp decline in survival between 500 and 800 mg/L suggests a critical toxicity threshold for zebrafish, underscoring the importance of concentration control in potential aquatic exposure scenarios.

### 2.5. Insecticidal Activity of AZA and AZA@MSNs–NH_2_ Against Helicoverpa armigera

AZA exhibits well-documented antifeedant, stomach toxic, contact toxic and growth-suppressive activities against a wide spectrum of insect species, especially lepidoptera pests [56]. In this work, we compared the insecticidal performance of free AZA, blank MSNs, and AZA@MSNs–NH_2_ against *H. armigera*, aiming to clarify how nanoencapsulation alters AZA’s pest control efficacy. As previously documented by Dawka et al. [16], AZA triggers insect toxicity via multi-target pathways: it accumulates inside the midgut of *H. armigera* with limited metabolic breakdown, thereby triggering feeding deterrence, suppressed larval growth, developmental malformations, and ultimately larval death.

#### 2.5.1. The Toxicity of AZA to *Helicoverpa armigera*

To compare the insecticidal toxicity of free AZA and AZA@MSNs–NH_2_, bioassays against *H. armigera* larvae were conducted. Blank control groups yielded average mortality rates lower than 5% across all tested concentrations (Table S2), implying that such minor mortality stemmed from random experimental noise rather than pesticide activity.

**Figure 6:**
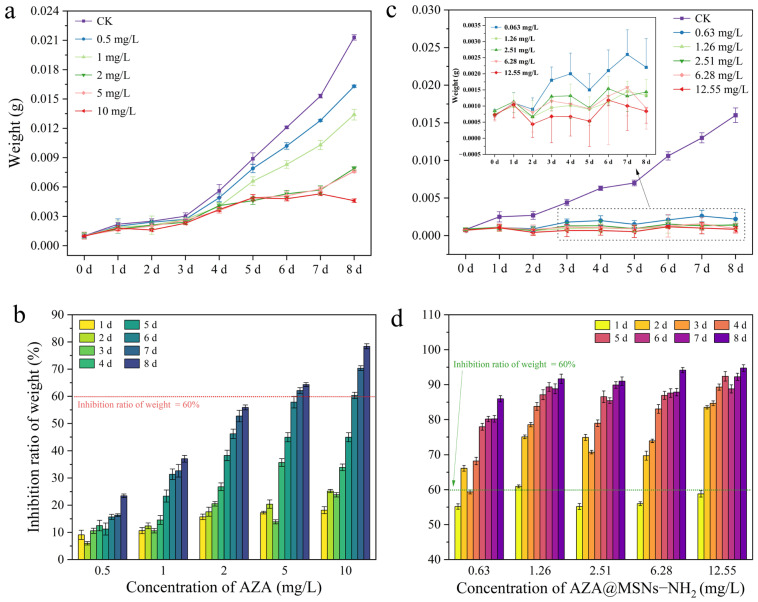
Growth inhibitory effects of free AZA and AZA@MSNs–NH_2_ against *Helicoverpa armigera*. Variations in larval body weight for AZA-treated groups (**a**); weight gain inhibition rates for AZA-treated groups (**b**); variations in larval body weight for AZA@MSNs–NH_2_-treated groups (**c**); weight gain inhibition rates for AZA@MSNs–NH_2_-treated groups (**d**).

AZA did not produce obvious suppression of larval weight gain within the first 3 days post-treatment (Figure 6a,b), consistent with its recognized delayed insecticidal mode of action [57]. Nevertheless, from day 4 to day 8, larvae receiving AZA treatments possessed markedly lower average body weights compared with the untreated control, displaying clear dose-dependent growth suppression (Figure 6a). At concentrations ≤ 2 mg/L, the maximum weight-growth inhibition rate hit 55.91 ± 0.90% on day 8. By contrast, higher concentrations of 5 and 10 mg/L induced over 60% growth inhibition as early as day 7, with the 10 mg/L group achieving an inhibition rate of 78.40 ± 0.92% at day 8.

Collectively, these data confirm that within the concentration range tested (≤10 mg/L), AZA functions primarily as a larval growth suppressor against *H. armigera*, instead of a fast-acting lethal pesticide.

#### 2.5.2. The Toxicity of MSNs to *Helicoverpa armigera*

To evaluate the potential inherent toxicity of MSNs against *H. armigera*, two bioassay methods—diet incorporation and insect dipping—were employed (Table S2). Across all treatment groups, the average mortality was less than 5% (i.e., fewer than one death per replicate), confirming that MSNs at concentrations up to 1000 mg/L are non-lethal to *H. armiger*.

#### 2.5.3. The Toxicity of AZA@MSNs–NH_2_ to *Helicoverpa armigera*

The enhanced bioactivity of AZA@MSNs–NH_2_ against *H. armigera* was evidenced by both growth inhibition and direct lethality (Figure 6c,d and Table S3). Throughout the 8-day post-treatment period, larvae in all nanoformulation-treated groups exhibited consistently lower body weights than the control, with no significant weight progression observed (Figure 6c), translating to growth inhibition rates of >60% at day 3 and >80% at day 8 (Figure 6d). Moreover, the nanoformulation induced substantial mortality, achieving a corrected mortality of 62.65% by day 8 (Table S3). This dual efficacy stands in stark contrast to free AZA, which at comparable concentrations acts primarily as an antifeedant with negligible lethal activity. The transition from pure growth inhibition to combined inhibitory-lethal action suggests that MSNs-mediated delivery not only improves AZA bioavailability but may also alter its pharmacokinetic profile, facilitating greater internal exposure and consequently eliciting stronger physiological responses in target insects, as demonstrated by Benítez et al. [34].

The impact of AZA@MSNs–NH_2_ on the antioxidant defense system of *H. armigera* was assessed by monitoring the dose-dependent activities of three key enzymes—CAT, POD, and SOD (Figure S8). Notably, CAT activity displayed a hormesis-like pattern, with inhibition at low concentrations followed by activation at higher doses, suggesting its role as an early sensor of oxidative challenge. POD, in contrast, was invariably suppressed throughout the tested concentration range, implying a more vulnerable enzymatic component. SOD activity decreased initially (at 5 mg) but exhibited a progressive recovery as the concentration increased, nearly reaching control values at 100 mg—a rebound that likely reflects a compensatory upregulation mechanism. The distinct responsiveness among these enzymes indicates that AZA@MSNs–NH_2_ imposes a substantial oxidative burden on *H. armigera* larvae; the inability of POD to recover, coupled with the delayed but partial restoration of SOD, ultimately overwhelms the coordinated antioxidant network and compromises the insect’s capacity to counteract oxidative damage. This systemic disruption of the antioxidant network likely underlies the growth inhibition and lethality observed in bioassays, as oxidative injury to midgut cells would impair nutrient absorption and metabolic homeostasis, consistent with the multi-target oxidative toxicity of azadirachtin toward cotton bollworm larvae reported by Dawkar, V. V. et al. [16].

### 2.6. Sublethal Toxicity of AZA and AZA@MSNs–NH_2_ Against Apolygus lucorum (Meyer-Dür)

Previous work has shown that neem oil induces strong avoidance and inhibits nymphal emergence in *Lygus hesperus* (Hemiptera, Miridae) [16], indicating significant behavioral and sublethal toxicity toward mirid bugs. Reasoning that AZA—the major active ingredient of neem oil—would exhibit similar activity, we evaluated the toxicity of AZA, MSNs, and AZA@MSNs–NH_2_ against *A. lucorum* (Meyer-Dür), aiming to determine how nano-encapsulation influences its bioefficacy against this hemipteran pest.

#### 2.6.1. The Toxicity of AZA to *Apolygus lucorum* (Meyer-Dür)

The lethal activity of AZA against *A. lucorum* was concentration- and time-dependent (Table S4). At concentrations ≤ 50 mg/L, mortality remained low throughout the 4-day observation period. In contrast, at 250 mg/L, mortality rose to 75 ± 0.78% by day 3. When the concentration was further increased to 250–300 mg/L, the insecticidal effect became both more rapid and more pronounced, with >60% mortality recorded at day 2 and complete mortality (100%) achieved by day 3. These results demonstrate that elevated AZA concentrations not only amplify the ultimate mortality rate but also shorten the time required to reach lethal endpoints, underscoring the critical role of dosage in optimizing the bioefficacy of AZA against *A. lucorum*.

#### 2.6.2. The Toxicity of MSNs to *Apolygus lucorum* (Meyer-Dür)

The inherent toxicity of MSNs to *A. lucorum* was assessed as a control (Table S4). During the initial 24 h, no mortality was observed across the entire concentration range. With extended exposure (3–4 days), however, a mild but detectable toxic effect emerged at elevated concentrations: at 500 and 800 mg/L, mortality reached 30% ± 5% by day 3, and at 800 mg/L, it rose to 42.5% ± 0.85% by day 4. This delayed and moderate toxicity at high doses confirms that MSNs themselves are not acutely lethal to *A. lucorum*, but may impose sublethal stress under prolonged exposure—a factor to be considered when evaluating the net contribution of the encapsulated AZA to the overall insecticidal activity of the nanoformulation.

#### 2.6.3. The Toxicity of AZA@MSNs–NH_2_ to *Apolygus lucorum* (Meyer-Dür)

As shown in Table S4, exposure to 62.75 mg/L AZA@MSNs–NH_2_ resulted in a 75 ± 1.31% mortality rate of *A. lucorum* after 4 days. At 125.5 mg/L, 60% mortality was attained within 3 days, while the 188.75 mg/L group reached 60 ± 1.25% mortality after only 2 days. For higher concentrations of 251 mg/L and 313.75 mg/L, 60% mortality was also recorded within 2 days. Moreover, treatments at 188.75, 251 and 313.75 mg/L all caused 100% mortality by day 3. These data reveal that AZA@MSNs–NH_2_ possesses prominent insecticidal activity against *A. lucorum* even at relatively low concentrations, though it delivers slow-acting efficacy. In contrast, this nanoformulation exerts fast and highly toxic effects at elevated concentrations.

## 3. Materials and Methods

### 3.1. Materials

Tetraethyl orthosilicate (TEOS), Hexadecyl trimethyl ammonium Bromide (CTAB), and (3-Aminopropyl) triethoxysilane (APTES) were purchased from Macklin Biochemical Technology Co., Ltd. (Shanghai, China). Methanol (≥99.9%), ethanol (≥99.9%), acetonitrile (≥99.9%), Dimethyl sulfoxide (DMSO, ≥99%), etc., were purchased from Sinopharm Chemical Reagent Co., Ltd. (Beijing, China). All water used in the research was purified water supplied by Wahaha Group Co., Ltd. (Hangzhou, China). Azadirachtin (AZA, 37%) was provided by South China Agriculture University as a friendly gesture(Guangzhou, China).

An intelligent artificial climate incubator (Model RXM-258A, 258 L) was supplied by Ningbo Jiangnan Instrument Factory (Ningbo, China).

Cotton (Yuanmian 8) and wheat (Liangchun 1758) seeds were used for cultivating seedlings in pot experiments (Urumqi, China). Zebrafish were purchased from Mingzhu Flower Market (Urumqi, China).

### 3.2. Methods

#### 3.2.1. Fabrication and Amination Modification of MSNs

MSNs were synthesized via a modified Sol–Gel method adapted from He et al. [58]. Briefly, CTAB (3.0 g) was dissolved in 500 mL of methanol–water (1:9, *v*/*v*) solution, followed by the addition of 8 mL of NaOH solution (2 mol/L). The mixture was stirred at room temperature for 30 min. Separately, TEOS (4 mL) was dispersed in methanol (16 mL) and then added dropwise to the above solution under continuous stirring. After reaction at room temperature for 8 h, the resulting white precipitate was collected by centrifugation (5000 rpm, 8 min), washed three times with ethanol and deionized water, and dried in an oven. Two template removal methods—calcination and acidic ethanol reflux—were subsequently employed to eliminate CTAB, and the microstructural properties of the resulting MSNs were compared (see Supplementary Materials for detailed methods).

Amino modification was carried out following a previously reported protocol [59]. Briefly, MSNs (150 mg) were dispersed in deionized water (120 mL), and the suspension was heated to 80 °C. APTES (120 μL) was then introduced, and the reaction was allowed to proceed for 8 h under stirring. After cooling to room temperature, the product was collected by centrifugation (5000 rpm, 8 min), washed three times with ethanol and deionized water, and dried in an oven.

#### 3.2.2. Preparation of AZA@MSNs–NH_2_

MSNs–NH_2_ and AZA were mixed at mass ratios of 20:10, 20:15, 20:17, 20:25, and 20:30 (total mass: 70 mg) in 180 mL of acetonitrile in a brown flask. Each mixture was sonicated for 30 min in the dark and then stirred for 24 h. The loading capacity of AZA was compared across the five formulations to identify the optimal feeding ratio.

#### 3.2.3. Assay for Photodegradation Resistance of AZA@ MSNs–NH_2_

AZA (2 mg) and AZA@MSNs–NH_2_ (6 mg) were separately dissolved in 4 mL DMSO and each solution was transferred into a corresponding quartz cuvette. The two quartz cuvettes were exposed to a UV germicidal lamp (36 W; wavelength: 254 nm) at a distance of 20 cm. At predetermined time intervals, the samples were analyzed using a UV–visible spectrophotometer [32]. The AZA content and degradation rate were calculated using the following equation.

Degradation rate (%) = (Content_−AZA−0_ − Content_−AZA−n_)/Content_−AZA−0_ × 100% (1)

where “Content_−AZA−0_” represents the initial AZA content in the DMSO suspension of AZA or AZA@MSNs–NH_2_; “Content_−AZA−n_” represents the AZA content in the DMSO suspension of AZA or AZA@MSNs–NH_2_ at a specific time interval.

#### 3.2.4. Biological Toxicity Tests on Plant Seeds, Seedlings and Zebrafish

Germination assays were carried out in triplicate using 90 mm Petri dishes lined with 85 mm filter paper. A stock solution of MSNs (1000 mg/L) was prepared by dispersing 10 mg of MSNs in 10 mL of deionized water, and serial dilutions were made to obtain working concentrations of 0.1, 1, 5, 10, 20, and 50 mg/L. Cotton seeds were surface-sterilized with 75% ethanol for 20 min, rinsed thoroughly with deionized water, and then placed (20 seeds per dish) onto filter paper moistened with 10 mL of each working solution. The Petri dishes were incubated at 25 ± 1 °C in the dark for 12 days, during which germination was monitored daily and seedling height was recorded at the end of the incubation period. The seed germination rate (GR_−cotton_, %) and germination potential (GP_−cotton_, %) were calculated using Equations (2) and (3).

GR_−cotton_ = G_−cotton−12_/G_−cotton−0_ ×100%, (2)

GP_−cotton_ = G_−cotton−4_/G_−cotton−0_ ×100%, (3)

where G_−cotton−0_ represents the total number of cotton seeds, G_−cotton−4_ and G_−cotton−7_ represent the number of germinated seeds on day 4 and day 7, respectively.

Wheat seeds: The MSNs stock solution was serially diluted with deionized water to obtain working solutions at concentrations of 0.1, 1, 5, 10, and 20 mg/L. For each treatment, 10 mL of the respective working solution and 20 wheat seeds were placed in a Petri dish. The dishes were incubated at 25 ± 1 °C in darkness until the germination rate in the control group exceeded 90%. Germination rate (GR_−wheat,_ %) and germination potential (GP_−wheat,_ %) were calculated using Equations (4) and (5).

GR_−wheat_ = G_−wheat−n_/G_−wheat−0_ ×100%, (4)

GP_−wheat_ = G_−wheat−n/3_/G_−wheat−0_ ×100%, (5)

where G_−wheat−0_ represents the total number of wheat seeds, G_−wheat−n/3_ denotes the number of germinated seeds on day n/3, and G_−wheat−n_ stands for the number of germinated seeds when the germination rate of the control group exceeds 90%.

Wheat seedlings: Plump and healthy wheat seeds were selected and germinated. Uniform seedlings with shoot lengths of 1 ± 0.2 cm were transferred to seedling trays at a density of 10 plants per group and treated with MSNs working solutions at 0.1, 1, 5, 10, and 20 mg/L. After 20 days of cultivation, growth parameters including root length, leaf length, and plant height were measured.

Zebrafish: Healthy adult zebrafish of uniform body size were acclimatized in the laboratory for 3 days prior to exposure. Groups of 10 fish were placed in plastic containers containing 100 mL of MSNs working solutions at 0.1, 1, 5, 10, and 20 mg/L. Mortality was recorded at 6, 12, 24, 48, and 96 h post-exposure, and survival rates were calculated accordingly.

#### 3.2.5. Determination of Physiological and Biochemical Indexes in Cotton and Wheat Seeds

The contents of gibberellin (GA) and abscisic acid (ABA) were determined using commercial enzyme-linked immunosorbent assay (ELISA) kits. GA was measured by the double-antibody sandwich method, and ABA was measured by the competitive ELISA method; absorbance was detected at 450 nm using a microplate reader.

α-amylase activity was determined using the 3,5-dinitrosalicylic acid colorimetric method at 540 nm. Trypsin activity was determined using the BAPNA chromogenic method at 405 nm.

Seed samples were homogenized in ice-cold buffer, centrifuged at 12,000 rpm for 10 min at 4 °C, and the supernatants were collected for analysis. All procedures were performed strictly according to the manufacturer’s instructions.

#### 3.2.6. Toxicity Determination of AZA, MSNs and AZA@ MSNs–NH_2_ to *Helicoverpa armigera*

Incubator conditions: Intelligent artificial climate incubator (Model RXM-258A, 258 L, Ningbo Jiangnan Instrument Factory, Ningbo, China), temperature 27 ± 2 °C, relative humidity 70 ± 5%, photoperiod 14L:10D. Artificial diet for *H. armigera* larvae was purchased from Keyun Biotechnology Co., Ltd. (Jiyuan, China), which was used for continuous rearing of test insects under laboratory conditions. Fifteen *H. armigera* larvae were used per replicate, with three replicates per treatment. Each larva was reared individually. Every treatment was exposed to different concentrations of free AZA, MSNs, and AZA@MSNs–NH_2_.

Feeding method: A predetermined mass of MSNs was thoroughly mixed with 16 g of artificial diet to prepare toxic diets with final concentrations of 1, 10, 20, 50, and 100 mg/g. The treated diet was placed into clean 5 mL glass vials, and one 4 h-starved third-instar *H. armigera* larva was introduced per vial. Survival of *H. armigera* was recorded at 24, 48, and 96 h.

Insect-dipping method: 20 mg of MSNs was accurately weighed and dispersed in 20 mL of deionized water, sonicated for 30 min to prepare a 1000 mg/L stock solution, and serially diluted to working solutions at 1, 10, 20, 50, and 100 mg/L. Third-instar larvae of *H. armigera* were immersed in each working solution for 30 s, removed, and blotted dry. Once dried, larvae were transferred to 5 mL glass vials containing diet (one larva per vial) and incubated. Survival was recorded at 24, 48, and 96 h.

Leaf-disk method: A certain amount of AZA and AZA@MSNs–NH_2_ were accurately weighed and dissolved in DMSO to prepare standard stock solutions: 500 mg/L for AZA and 122.5 mg/L for AZA@MSNs–NH_2_ (calculated based on AZA content). The AZA stock solution was serially diluted to 0.5, 1, 2, 5, and 10 mg/L working solutions. The AZA@MSNs–NH_2_ stock solution was serially diluted to 1.63, 1.26, 2.51, 6.58, 12, and 55 mg/L working solutions. Circular leaf discs (1.5 cm diameter) were punched from fresh cabbage leaves, immersed in each working solution for 30 s, removed, and air-dried. Treated leaf discs were placed in 60 mm petri dishes (two discs per dish), and one 4 h-starved third-instar *H. armigera* larva was introduced per dish. Larvae were incubated for 8 days; larval weight was recorded daily, and survival and mortality were calculated. Leaf discs were replaced every 2 days.

#### 3.2.7. Measurement of Antioxidant Enzyme Activities in *Helicoverpa armigera*

Antioxidant enzyme activities, including superoxide dismutase (SOD), catalase (CAT), and peroxidase (POD), were determined using commercial microplate-based assay kits. Briefly, *H. armigera* tissue samples were homogenized in ice-cold extraction buffer and centrifuged at 12,000 rpm for 10 min at 4 °C. The supernatant was collected for subsequent analysis.

SOD activity was measured using the WST-8 method at 450 nm with a microplate reader.

CAT activity was determined by monitoring the decomposition of H_2_O_2_ at 510 nm.

POD activity was assayed using the guaiacol colorimetric method at 470 nm.

All procedures were performed strictly following the manufacturer’s instructions, and enzyme activities were calculated based on tissue fresh weight.

#### 3.2.8. Toxicity Determination of AZA, MSNs and AZA@ MSNs–NH_2_ to *Apolygus lucorum*

Incubator parameters: Intelligent artificial climate incubator (Model RXM-258A, 258 L, Ningbo Jiangnan Instrument Factory, Ningbo, China), temperature 26 ± 1 °C, relative humidity (75 ± 5) %, photoperiod L:D = 16 h:8 h. Twenty *A. lucorum* individuals were used per experimental group, with three replicates per group. Individuals that showed no movement when touched with tweezers were recorded as dead.

MSNs (topical application method): A predetermined mass of MSNs was accurately weighed and dispersed in deionized water containing 2% Tween-80 to prepare a 1000 mg/L standard stock solution. The stock solution was serially diluted to working solutions of 50, 100, 200, 500, and 800 mg/L. A 1 μL microsyringe was used to apply each working solution onto the thoracic dorsum of *A. lucorum*. Treated insects were transferred individually into finger tubes. A 0.2% Tween-80 aqueous solution was used as the control. Cowpea pods were provided as routine food, and mortality was recorded at 24, 48, 72, and 96 h after incubation.

AZA and AZA@MSNs–NH_2_ (diet-incorporation bioassay): Predetermined amounts of AZA and AZA@MSNs–NH_2_ were accurately weighed and dissolved in DMSO to prepare standard stock solutions of 1000 mg/L (AZA) and 1225 mg/L (AZA@MSNs–NH_2_, based on AZA content), respectively. The AZA stock solution was serially diluted with 2% Tween-80 aqueous solution to working solutions of 50, 150, 200, 250, and 300 mg/L. The AZA@MSNs–NH_2_ stock solution was serially diluted with 2% Tween-80 aqueous solution to working solutions of 62.75, 125.5, 188.75, 251, and 313.75 mg/L. Cowpea (*Vigna unguiculata*) was cut into 7 cm strips, immersed in each working solution for 30 s, and then air dried. The control group was treated with 0.2% Tween-80 aqueous solution. Treated cowpea strips were placed into ventilated finger tubes, and twenty *A. lucorum* nymphs were introduced into each tube. After incubation for 24, 48, and 72 h, mortality was recorded and calculated.

### 3.3. Statistical Analysis

Experimental data were processed and statistically analyzed with Microsoft Office Excel LTSC 2024 and SPSS 26.0. All graphs were plotted using Origin 2024 and Microsoft Office PowerPoint LTSC 2024. All measurement results were expressed as mean ± standard error. There are significant differences between the numbers marked with different letters (*p* < 0.05). Duncan’s multiple range test was used for multiple-group comparisons. Values labeled with identical lowercase letters indicated no significant intergroup differences, whereas different letters denoted significant differences at *p* < 0.05.

## 4. Conclusions

In summary, this study has established an MSNs-based nanocarrier system that effectively addresses the intrinsic physicochemical shortcomings of AZA while substantially enhancing its insecticidal performance against two major cotton pests. Through systematic optimization of MSNs synthesis—including NaOH concentration and template removal strategy—high AZA loading capacity was achieved. The bioassay results demonstrated that AZA@MSNs–NH_2_ nanoformulation possesses three key attributes: (i) markedly improved photostability, with only 25.76 ± 3.33% and 63.00 ± 1.53% degradation after 6 and 24 h of UV irradiation; (ii) favorable biosafety, as evidenced by low-concentration promotion of cotton germination/growth and negligible zebrafish toxicity; and (iii) superior bioactivity, with potent growth inhibition and lethality against *H. armigera* and moderate efficacy against *A. lucorum*. Collectively, this work not only expands the application horizon of AZA but also provides a technically simple and environmentally benign strategy for sustainable pest management in cotton cultivation, highlighting the considerable potential of MSNs-based nanoinsecticides for further development and field implementation.

## Data Availability

All data generated or analyzed as part of this study are available in the article.

## Supplementary Materials

The following supporting information can be downloaded at: https://www.mdpi.com/article/10.3390/molecules31132347/s1, Figure S1: Nitrogen adsorption-desorption isotherms and pore size distributions (insets) of (a) MSNs and (b) AZA@MSNs−NH_2_; Figure S2. Detection of the Zeta potential of MSNs and AZA@MSNs−NH_2_; Figure S3. UV-Vis (a) and LC (b) spectra of AZA@MSNs; Figure S4. The TEM images of MSNs (a) and AZA@MSNs−NH_2_ (b); Figure S5. The AZA@MSNs−NH_2_’s spectra as well as quantitative curve and equation of LC (a) and UV-Vis (b); Table S1: The effects of MSNs on cotton and wheat seeds and seedlings; Figure S6. The ABA (a), GA (b), α-AL (c) and trypsin activities (d) of wheat seeds and cotton seeds; Figure S7. The survival rates of zebrafish treated with different concentration of MSNs; Table S2. The death rate of *Helicoverpa armigera* after treated with different concentrations of AZA and MSNs through two methods; Table S3. The death rate of *Helicoverpa armigera* after treated with different concentrations of AZA@MSNs−NH_2_; Figure S8. The enzymatic activities of catalase (CAT), peroxidase (POD), and superoxide dismutase (SOD) in *Helicoverpa armigera*; Table S4. The death rate of *Apolygus lucorum* (Meyer-Dür) after treated with different concentrations of MSNs, AZA and AZA@MSNs−NH_2_ [60,61].
