# Supplementary material for: Nanoformulation of Azadirachtin Improves Its Control on Cotton Pests
Source: Molecules. 2026 Jul 3;31(13):2347. doi: 10.3390/molecules31132347 (PMC13363519; doi:10.3390/molecules31132347)
Supplement: Supplementary file 1 [file molecules-31-02347-s001.zip › molecules-4421656-supplementary.pdf]

Supplementary Materials

Nanoformulation of azadirachtin improves its control on cotton pests

Zhiwei Tang [1,2], Jianhao Dong [1,2], Yue Sun [1], Chuhela Tabusibieke [1], Yujiao Wang [1,2*] and Wei Lu [1,2,*]

1 Key Laboratory of the Pest Monitoring and Safety Control of Crops and Forests of the Universities of the Xinjiang Uygur Autonomous Region, College of Agronomy, Xinjiang Agricultural University, No. 311, Nongda East Road, Sayibak District,
Urumqi 830052, China; 18320027289@163.com (Z.T.); dong371014@163.com (J.D.); 15709013626@163.com (Y.S.); c21521sahpr@foxmail.com (C.T.)

2 Engineering Research Centre of Cotton, Ministry of Education, Urumqi 830052, China

* Correspondence: yujiaowang@xjau.edu.cn (Y.W.); teerakon@sina.com (W.L.)

S1. Methods

S1.1. Removing templates

Reflux: Disperse the as-synthesized MSNs into 150 mL of ethanol. Subsequently, 400 μL of 1 mol/L HCl was added. The mixture was refluxed for 6 hours, and this process was repeated twice [1].

Calcination: The as-synthesized MSNs were placed in a crucible and calcined in a muffle furnace at 550°C for 5 hours [2]. The muffle furnace (ceramic fiber box-type resistance furnace, model: SX2-4-10LTP) was purchased from Hunan Lichen Instrument Technology Co., Ltd.

S1.2. Characterization of MSNs−HN_2, AZA@ MSNs−NH_2

X-ray diffraction (XRD) patterns were recorded using a Bruker D8 Advance diffractometer equipped with a LynxEye XE-T detector under 2θ scanning mode. The particle size distribution of MSNs was determined using a PSS A7000 APS-DLS analyzer. The morphology of MSNs−NH_2 and AZA@MSNs−NH_2 was characterized via scanning electron microscopy (SEM SU8020; HITACHI, Japan). Fourier transform infrared (FT-IR) spectra were acquired using an IRtrace-100 FT-IR spectrometer (Shimadzu, Japan). UV-Vis absorption spectra were measured using a Cary 300 UV-Vis Spectrophotometer (Agilent Technologies, USA). All measurements were performed in triplicate.

Liquid chromatography (LC) analysis was performed on a Waters LC system equipped with a reversed-phase Diones C18 chromatographic column (250 mm × 4.6 mm, 5 μm) and a UV-Vis detector, operating at 218 nm at room temperature. The flow rate and injection volume were set at 0.8 mL/min and 10 μL, respectively. The mobile phase was composed of methanol/water (80:20, v/v). Under these experimental conditions, the retention time of azadirachtin (AZA) was 7.119 min [3].

Zeta potential was determined using a Zetasizer instrument (Malvern Panalytical, Malvern Instruments Ltd). Measurements were carried out at 25 °C with deionized water as the dispersant (refractive index: 1.330, viscosity: 0.8872 cP, dielectric constant: 78.5). A clear disposable zeta cell was used, and each sample was tested with 10 runs. Data were processed using Zetasizer software (Ver. 8.01.4906), and the mean zeta potential value was reported.

Specific surface area, pore volume, and pore size distribution were measured using a TriStar II 3020 surface area and porosity analyzer (Micromeritics, Norcross, GA, USA). Samples were degassed under vacuum before analysis. Nitrogen adsorption–desorption isotherms were recorded at −195.8 °C. The BET (Brunauer–Emmett–Teller) method was used to calculate specific surface area. Pore structure parameters were obtained via the BJH (Barrett–Joyner–Halenda) model and t-plot method, covering a pore diameter range of 1.7–300 nm.

High-resolution transmission electron microscopy (HRTEM) images were obtained using a field-emission transmission electron microscope (FEI Tecnai G2 F20) operated at an accelerating voltage of 200 kV. The sample was dispersed in ethanol, dropped onto a copper (Cu) grid with a carbon support film, and dried naturally before observation.

S1.3. Loading Rate and Encapsulation Efficiency of AZA@MSNs−NH_2

AZA stock solution (1000 mg/L) was prepared by dissolving 0.0541 g AZA (purity: 37%) in 20 mL acetonitrile. The stock solution was serially diluted to prepare working standard solutions at concentrations of 20, 50, 100, 200, 500, and 800 mg/L. Each standard solution was characterized via LC and UV–Vis spectroscopy, and calibration curves were established by plotting LC peak area or UV–Vis absorbance versus AZA concentration (Figure S5).

Accurately weigh 5 mg of AZA@MSNs−NH_2 and disperse it in 5 mL of DMSO (the theoretical concentration of AZA, $C_{-theoretical}$ = 1000/2.25 mg/L). After making the AZA@MSNs−NH_2 solution under ultrasonication for 30 minutes detect with HPLC and UV-Vis respectively. Substitute the measured results into the corresponding linear equation to obtain the actual concentration of AZA (C_{-test}). The loading capacity of AZA is calculated according to the following formula (1).

$$\text{Loading rate of AZA (\%)} = (C_{-test} / C_{-theoretical}) \times 100\%, \quad \text{(S1)}$$

Cumulative release profiles: A 3 mL aliquot of AZA@MSNs−NH_2 suspension (500 mg/L nanoparticles) was dispersed in a quartz cuvette. The suspension was detected at predetermined time points via a UV–visible spectrophotometer at 273 nm. The measured absorbance values were substituted into the corresponding linear equation to determine the real-time AZA concentration (C_t), which was calculated using the following formula (2).

$$C_t = (A + 0.06839) / 0.00172, \quad \text{(S2)}$$

The initial AZA concentration (C_0 = 49.8159 mg/L) was calculated from the absorbance value at 0 h. The theoretical maximum release concentration (C_{max} = 10.6 mg/L) was calculated based on the encapsulation efficiency and nanoparticle concentration, representing the maximum AZA concentration after complete release of encapsulated drugs. The cumulative release rate of AZA was calculated using the following formula (3).

$$\text{Cumulative release rate of AZA (\%)} = (C - 49.815891) / 10.6 \times 100\%, \quad \text{(S3)}$$

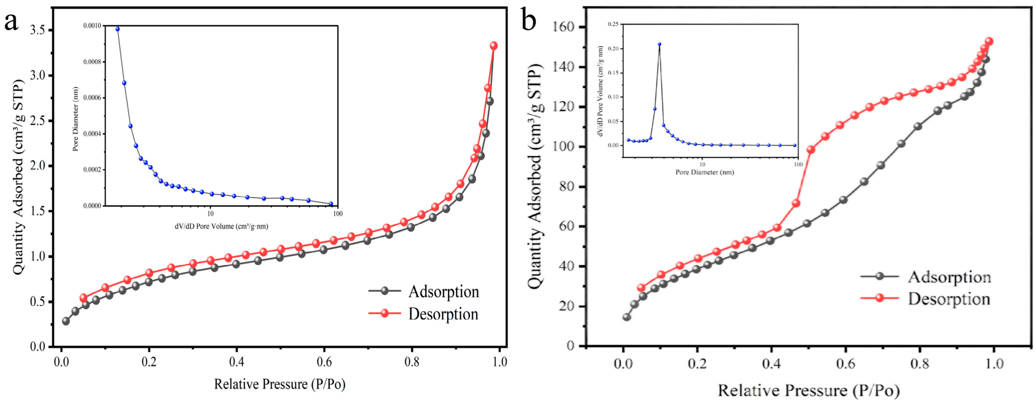

Figure S1. Nitrogen adsorption-desorption isotherms and pore size distributions (insets) of (**a**) MSNs and (**b**) AZA@MSNs−NH2.

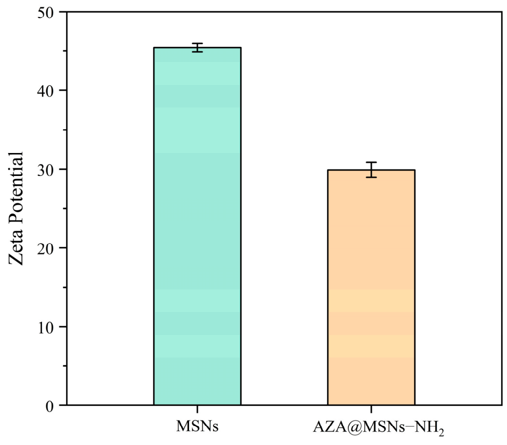

Figure S2. Detection of the Zeta potential of MSNs and AZA@MSNs−NH2.

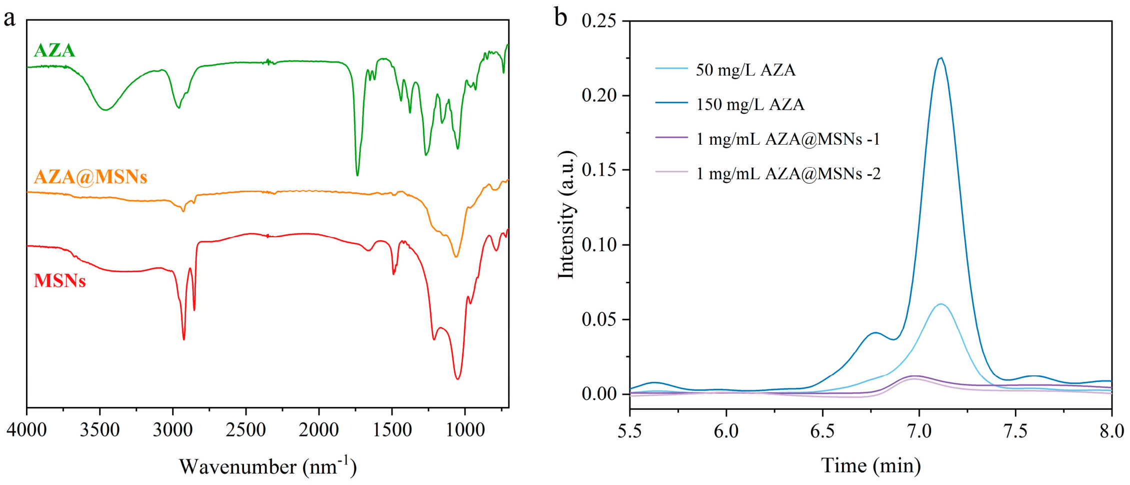

Figure S3. UV-Vis (**a**) and LC (**b**) spectra of AZA@MSNs.

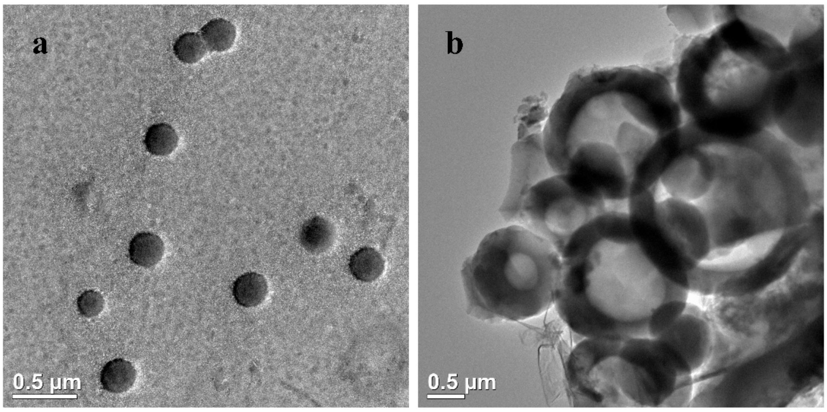

Figure S4. The TEM images of MSNs (a) and AZA@MSNs−NH_2 (b).

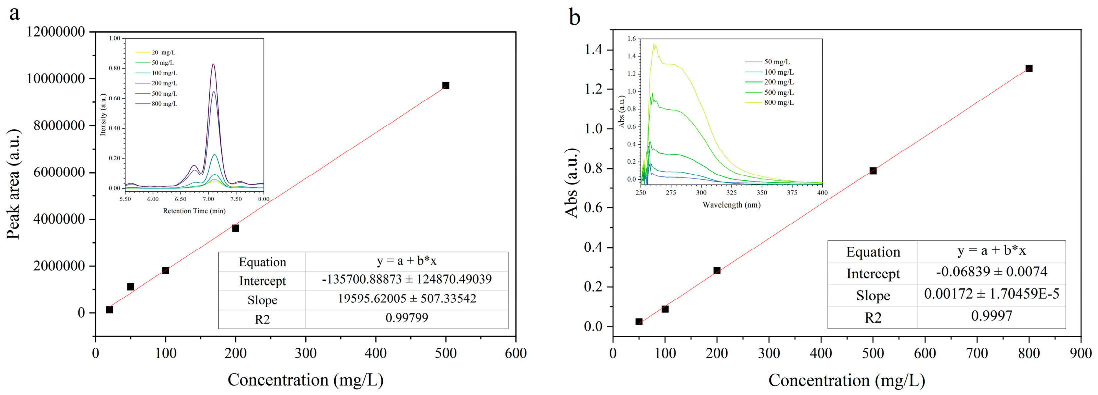

Figure S5. The AZA@MSNs−NH_2's spectra as well as quantitative curve and equation of LC (**a**) and UV-Vis (**b**).

Table S1. The effects of MSNs on cotton and wheat seeds and seedlings

	Cotton			Wheat				
	GR (%)	GP (%)	Height of seedling (cm)	GR (%)	GP (%)	Root length (cm)	Leaf length (cm)	Height of seedling (cm)
CK	54.00 ± 1.00 a	27.33 ± 2.31 b	6.52 ± 0.31 b	91.67 ± 2.89 b	70.00 ± 0.00 a	91.67 ± 2.89 a	17.1 ± 0.21 a	21.6 ± 0.23 a
50 mg/L	62.67 ± 1.53 a	43.33 ± 1.53 a	7.23 ± 0.14 a	93.33± 2.89 ab	71.67 ± 2.89 a	90.00 ± 0.00 a	16.8 ± 0.18 a	21.4 ± 0.12 a
100 mg/L	64.00 ± 1.00 a	46.67 ± 2.52 a	7.65 ± 0.20 a	96.67 ± 2.89 a	73.33± 5.78 a	86.67 ± 2.89 ab	16.2 ± 0.22 a	22.1 ± 0.12 a
200 mg/L	66.00 ± 2.00 a	46.00 ± 1.00 a	7.86 ± 0.17 a	78.33± 2.89 c	61.67 ± 2.89 b	75.00 ± 0.00 b	15.8 ± 0.18 b	21.9 ± 0.21 a
500 mg/L	71.33 ± 1.53 a	46.00 ± 2.00 a	7.52 ± 0.11 a	66.67 ± 2.89 d	38.33± 2.89 c	58.33± 2.89 c	12.0 ± 0.15 c	16.2 ± 0.15 b
800 mg/L	72.00 ± 2.65 a	50.00 ± 2.00 a	7.22 ± 0.13 a	50.00 ± 5.00 e	25.00 ± 5.00 d	43.33 ± 2.89 d	9.10 ± 0.09 d	13.5 ± 0.12 c
1000 mg/L	56.00 ± 2.00 a	32.67 ± 1.15 b	6.60 ± 0.35 b	/	/	/	/	/

Note: There are significant differences between the numbers marked with different letters ($P < 0.05$).

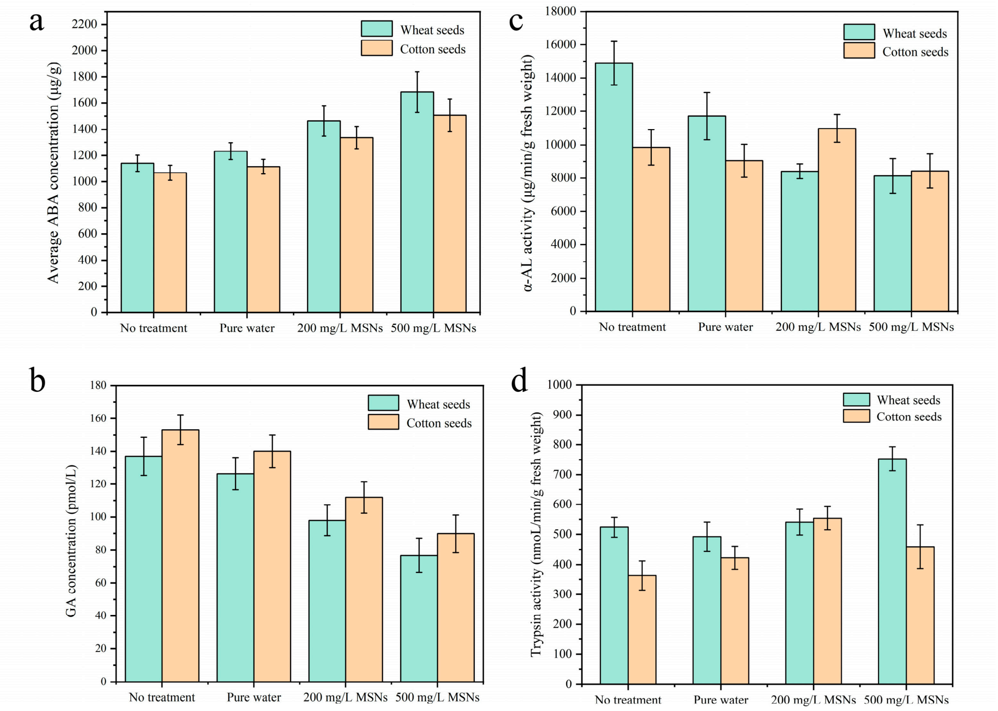

Figure S6. The ABA (**a**), GA (**b**), α-AL (**c**) and trypsin activities (**d**) of wheat seeds and cotton seeds.

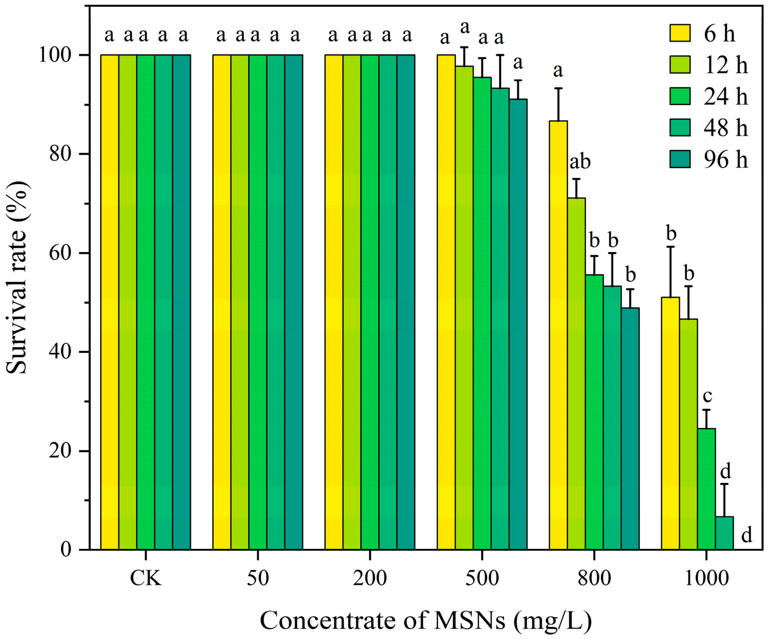

Figure S7. The survival rates of zebrafish treated with different concentration of MSNs.

Table S2. The death rate of *Helicoverpa armigera* after treated with different concentrations of AZA and MSNs through two methods.

	Concentration	Average rate of death ± SD (%)		
	(mg/L)	24 h	48 h	96 h
AZA	CK	0 ± 0	0 ± 0	0 ± 0
	0.5	0 ± 0	0 ± 0	0 ± 0
	1	2.22 ± 0.58	2.22 ± 0.58	2.22 ± 0.58
	2	0 ± 0	0 ± 0	0 ± 0
	5	0 ± 0	0 ± 0	0 ± 0
	10	0 ± 0	0 ± 0	0 ± 0
MSNs:	CK	0 ± 0	0 ± 0	0 ± 0
Poisonous feed	10	0 ± 0	0 ± 0	0 ± 0
feeding method	20	0 ± 0	0 ± 0	0 ± 0
	50	0 ± 0	0 ± 0	0 ± 0
	100	0 ± 0	0 ± 0	0 ± 0
	500	0 ± 0	2.22 ± 0.58	2.22 ±0.58
	1000	0 ± 0	0 ± 0	0 ± 0
MSNs:	CK	0 ± 0	0 ± 0	0 ± 0
Insect immersion	1	0 ± 0	0 ± 0	0 ± 0
method	10	0 ± 0	0 ± 0	0 ± 0
	20	0 ± 0	0 ± 0	0 ± 0
	50	0 ± 0	0 ± 0	0 ± 0
	100	0 ± 0	0 ± 0	0 ± 0

Table S3. The death rate of *Helicoverpa armigera* after treated with different concentrations of AZA@MSNs−NH_2.

	48 h	72 h		96 h		8 d	
	Death rate ± SD	Death rate ± SD	Corrected death rate	Death rate ± SD	Corrected death rate	Death rate ± SD	Corrected death rate
CK	0.00 % ± 0	13.00% ± 0	/	13.00% ± 0	/	16.67% ± 0.14	/
0.63	8.76% ± 0.08	21.00% ± 0.07	9.20%	21.00% ± 0.07	9.20%	33.67% ± 0.08	20.08%
1.26	12.67% ± 0.13	21.33% ± 0.14	9.58%	21.33% ± 0.14	9.58%	44.00% ± 0.08	32.53%
2.51	17.00% ± 0.19	25.00% ± 0	13.79%	33.33% ± 0.14	23.37%	56.50% ± 0.09	47.59%
6.28	21.00% ± 0.19	38.00% ± 0	28.74%	54.67% ± 0.14	47.89%	63.00% ± 0.14	55.42%
12.55	37.76% ± 0.13	46.00% ± 0.19	37.93%	58.67% ± 0.08	52.49%	69.00% ± 0.08	62.65%

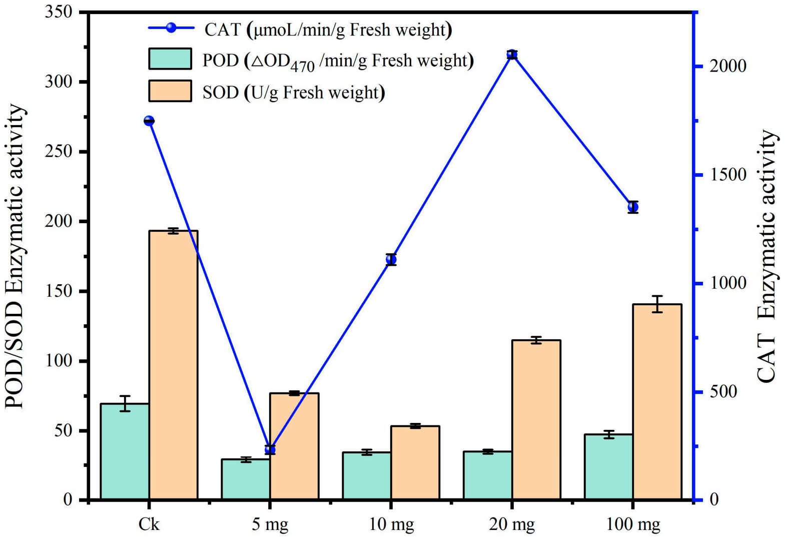

Figure S8. The enzymatic activities of catalase (CAT), peroxidase (POD), and superoxide dismutase (SOD) in *Helicoverpa armigera*.

Table S4. The death rate of *Apolygus lucorum* (Meyer-Dür) after treated with different concentrations of MSNs, AZA and AZA@MSNs–NH_2.

	Concentration	Rate of Death (%)			
	(mg/L)	24 h	48 h	72 h	96 h
MSNs	CK	0 ± 0	0 ± 0	0 ± 0	0 ± 0
	50	0 ± 0	0 ± 0	0 ± 0	0 ± 0
	100	0 ± 0	6.67 ± 0.58	10.00 ± 1.00	10.00 ± 1.00
	200	0 ± 0	5 ± 0.69	10 ± 1.06	17.5 ± 1.12
	500	0 ± 0	15 ± 1.06	25 ± 0.84	30 ± 0.69
	800	0 ± 0	20 ± 6.85	35 ± 1.64	42.5 ± 0.85
AZA	CK	0 ± 0	0 ± 0	0 ± 0	0 ± 0
	50	0 ± 0	0 ± 0	0 ± 0	10 ± 1.05
	150	7.5 ± 0.85	15 ± 0.46	32.5 ± 1.18	37.5 ± 1.25
	200	8.33 ± 1.44	42.5 ± 1.25	75 ± 0.78	100 ± 0
	250	25 ± 1.77	67.5 ± 1.06	100 ± 0	100 ± 0
	300	35 ± 1.63	82.5 ± 1.18	100 ± 0	100 ± 0
AZA@MSNs–NH_2	CK	0 ± 0	0 ± 0	0 ± 0	0 ± 0
	62.75	5 ± 0.65	17.5 ± 1.18	47.5 ±0.46	75 ± 1.31
	125.5	15 ± 0.46	35 ± 1.43	62.5 ± 0.97	89.5 ± 1.37
	188.75	25 ± 8.84	60 ± 1.25	100 ± 0	100 ± 0
	251	35 ± 0.46	77.5 ± 1.46	100 ± 0	100 ± 0
	313.75	47.5 ± 0.46	95 ± 0.85	100 ± 0	100 ± 0

Reference

1. Gai, X.; Yang, D.; Tang, R.; Luo, M.; Lu, P.; Xing, C.; Yang, R.; Ma, Q.; Li, Y. Preparation of Ni–Co/SiO_2 catalyst by ammonia reflux impregnation and its CH_4–CO_2 reforming reaction performance. *Fuel* 2022, 316, 123387.
2. Liu, J. Preparation and Performance Study of Amorphous SiO_2 Nanoparticles Under Different Catalytic Conditions. Master's Thesis, Lanzhou University, Lanzhou, China, 2022. (in Chinese)
3. Jin, D.; Ding, B.; Yong, D.; Gong, A.; Wang, Y. Determination of azadirachtin residues in vegetables by accelerated solvent extraction and HPLC. *Agrochemicals* 2020, 59, 52–55. (in Chinese)
